# Predicting Survey Responses: How and Why Semantics Shape Survey Statistics on Organizational Behaviour

**DOI:** 10.1371/journal.pone.0106361

**Published:** 2014-09-03

**Authors:** Jan Ketil Arnulf, Kai Rune Larsen, Øyvind Lund Martinsen, Chih How Bong

**Affiliations:** 1 Department of Leadership and Organizational Behaviour, BI Norwegian Business School, Oslo, Norway; 2 Management and Entrepreneurship Division, Leeds School of Business, University of Colorado at Boulder, Boulder, Colorado, United States of America; 3 Faculty of Computer Science and Information Technology, University of Malaysia at Sarawak, Sarawak, Malaysia; University of Memphis, United States of America

## Abstract

Some disciplines in the social sciences rely heavily on collecting survey responses to detect empirical relationships among variables. We explored whether these relationships were *a priori* predictable from the semantic properties of the survey items, using language processing algorithms which are now available as new research methods. Language processing algorithms were used to calculate the semantic similarity among all items in state-of-the-art surveys from Organisational Behaviour research. These surveys covered areas such as transformational leadership, work motivation and work outcomes. This information was used to explain and predict the response patterns from real subjects. Semantic algorithms explained 60–86% of the variance in the response patterns and allowed remarkably precise prediction of survey responses from humans, except in a personality test. Even the relationships between independent and their purported dependent variables were accurately predicted. This raises concern about the empirical nature of data collected through some surveys if results are already given *a priori* through the way subjects are being asked. Survey response patterns seem heavily determined by semantics. Language algorithms may suggest these prior to administering a survey. This study suggests that semantic algorithms are becoming new tools for the social sciences, opening perspectives on survey responses that prevalent psychometric theory cannot explain.

## Introduction

In this study, we explore how survey response patterns may be predicted using information available prior to conducting a survey. Such techniques have several interesting consequences for theory development and testing in the social sciences.

Many social science disciplines acquire data from surveys. The focus of interest is usually in how different variables relate to each other, allowing exploration of relationships such as those between leadership, motivation and work outcomes. To understand how these variables are related, researchers have hypothesised the existence of ‘latent variables’ – hidden sources of quantitative variation stemming from variables such as different types of leadership and motivation [1].

The Achilles heel of this research is the nature of variation in survey scores. The most common input to the computational tools is the inter-item correlation matrix, or the degree to which any two items in the survey tend to co-vary in a systematic way [2]. Commonly, the non-random patterns in survey responses are understood to reflect the systematic influence of some psychological or social variables on the respondents.

However, a fundamentally different explanation is possible. The main source of quantitative variation in the surveys may instead be the degree of semantic overlap among the items. We will attempt to show empirically how a *semantic theory of survey response* (STSR) allows an alternative interpretation of survey data from areas such as leadership, motivation and self-reported work outcomes, affecting views on theory formation, research methods and empirical data.

## A Theory of Semantic Survey Response

### Empirical, psychological, and semantic components of variance in survey data

The statistical treatment of survey data in the social sciences has developed as a discipline often referred to as ‘psychometrics’, originally developed from research on intelligence [3], [4]. Intelligence tests consist of (often non-verbal) tasks to be solved, and responses are recorded fairly objectively as ratings of error frequency or response speed, and are therefore not susceptible to semantically determined responses. Later, Rensis Likert introduced a method familiar to most people today – having respondents rate a statement on a scale from “strongly approve” to “strongly disapprove” or similar [5]. Seemingly akin to intelligence tests, this is something altogether different and the origins and nature of the recorded variance are debatable [6]–[8].

We cannot know *a priori* how a respondent will rate a given item, e.g. “I like to work here”. But once the respondent has chosen a value, the values for the next items may probably be given to some extent. To take an example: “Today is Monday”. Someone rating this as “very true” is very likely to give the same rating to “Tomorrow is Tuesday”. Most items are not as obviously linked. But someone affirming that “I like to work here” may with a similar probability endorse “I do not want to quit this job”.

This semantic linkage of items is the core of what we believe to be a misunderstanding in survey-based research, demonstrable through semantic research. General psychometric theory asserts that some semantic overlap is necessary to create intra-scale consistency, usually measured by the formula called ‘Cronbach's alpha’ [1]. But the semantic overlap needs to stop there. If the semantic overlap continues across scales, it is regarded as a contamination of the data since one scale will automatically correlate with another. To prevent this, prevalent psychometric practices call for statistical procedures called exploratory and confirmatory factor analysis (CFA). By convention, the proper conduction of such analyses is taken as proof that relationships among variables are empirical and not self-evident [9].

As we will show empirically, this assumption does not hold. The semantic relationships hold across different scales despite their apparent separation by factor analysis. The resulting inter-item correlations can be explained by their semantic relationships. This is unfortunate because it undermines the value of factor analysis in establishing scale independency and also raises fundamental questions about the empirical object of such techniques.

Our concerns are not new in research on surveys and psychometric theory. More than five decades ago, Coombs and Kao [10] demonstrated that factor analysis in itself will always produce an extra factor that they called the “social utility function”. This factor determines the data structure simply due to the meaning of the items, which all respondents would need to interpret in order to answer the survey. Coombs developed this function into a psychometric theory called “unidimensional unfolding”. As Coombs predicted, this has been shown to influence factor analyses [11], [12]. More importantly, experiments have shown that the quantitative properties of surveys are created by the semantic properties of items and their answering categories [8]. This may explain how independent research has shown respondents to provide responses where they in reality hold no opinion, or even to totally fictitious topics [6],[7].

The need of the digital community to store, search, index and extract large amount of texts has stimulated the development of techniques that are sufficiently reliable and developed to take on survey research [13]. The task at hand is theoretically straightforward: If the overlap of meaning between any two survey items can be estimated quantitatively, the estimate can be used to explore the degree to which respondents are actually answering according to what is semantically expected.

We have chosen two types of text algorithms for this task. One is called *latent semantic analysis* (LSA), which has previously been shown to perform very similarly to human language learning using large chunks of text as its input. The second type of algorithm is corpus-based, which means that it uses a lexical database and knowledge about sentence syntax structure as input. The one we use here will be referred to as ‘MI’, a term used by its developers (MI is just a name for the algorithm) [14], [15]. Both types of algorithm explore the semantic similarity of two different texts and return a measure expressing probable degree of semantic overlap. The team of authors has access to more advanced and efficacious techniques, but LSA and MI are used because they have been previously published, are well understood, allow easy replication, and remove uniqueness of algorithms as an explanation for our findings. We will refer to these two techniques together as *semantic analyses* and their numerical output as *semantic similarity indices*.

LSA functions by analysing texts to create a high-dimensional ‘semantic space’ in which all terms have specific locations, represented as vectors. LSA can then ‘understand’ new texts as combinations of term vectors in this space. LSA aggregates the word contexts in which a given word does/does not appear and provides a set of constraints that determines the similarity of meanings of words and sets of words. Thus, when two terms occur in contexts of similar meaning –even in cases where they never occur in the same passage –the reduced-dimension solution represents them as similar. Similarity is indicated by the cosines of the vectors in semantic space, taking on values between −1 and 1. Some practical examples: The two sentences “doctors operate on patients” and “physicians do surgery” have no words in common, but a commonly used LSA semantic space called TASA (Touchstone Applied Science Associates) estimates their overlap in meaning at .80. Furthermore, sentences with similar words do not necessarily appear as similar. For example, the LSA cosine for the two expressions “the radius of spheres” and “a circle's diameter” is .55, but the cosine for the sentence pair “the radius of spheres” and “the music of spheres” is only .01 [16].

LSA represents a sparse matrix of documents (columns) vs. terms-in-those-documents (rows). The matrix is generally set to downweigh common words. It is sometimes normalized before using an algorithm –singular value decomposition –similar to factor analysis. LSA then yields the aforementioned semantic space. This method now has well-documented text-recognition applications [17], [18]. LSA works across languages. It is viable in both research and commercial contexts, and it performs almost as well as humans on complex knowledge-management and integration tests [19]. The usefulness of this technique has been documented in determining identities of a wide range of constructs in the Information Systems discipline [13], [20].

Our approach was to let LSA detect accumulated knowledge and semantic relationships within texts relevant to respondents of organisational surveys. We defined relevant texts as articles from three different domains of media: Business-press texts, general newspaper texts, and PR-related texts.

The business-press texts were excerpts from *The Wall Street Journal*, *Business Week*, *Forbes* and *Fortune*. These excerpts covered a total of 84,836 texts from the years 1998–2007, covering a total of 45,816,686 words with 169,235 unique words.

The news excerpts were from *The New York Times*, *Los Angeles Times*, *Chicago Tribune*, *The Washington Post*, *The Boston Globe*, *USA Today*, *Houston Chronicle*, *San Francisco Chronicle* and *The Denver Post*. The years covered were again 1998–2007, including 162,929 texts covering 107,239,064 total words with 286,312 unique words.

The PR statements were taken from *PR Newswire*, covering the years 2003–2007. This sample included 212,484 texts with 151,450,055 total words and 423,001 unique words.

These materials allowed us to create three distinct ‘semantic spaces’, i.e. high-dimensional spaces in which all terms have a specific vector or location, allowing LSA to ‘understand’ the text of survey items. Every survey item in the study was projected into each semantic space to generate its mathematical representation (vector). These representations were, in turn, compared to each other, allowing computation of cosine angles between all the item vectors, with higher cosines indicating higher similarity between items. This procedure was repeated for all three semantic spaces.

While LSA ‘extracts’ meaning from the way words are used in texts, the MI algorithm [21] uses a lexical database called WordNet [22]–[24]. Briefly explained, MI derives the meaning of a text from knowledge already existing in WordNet [15], [21]. Similar to LSA, the MI algorithm produces an index of semantic identity ranging from 0 to 1. However, MI differs from LSA in that its values reflect only lexical knowledge encoded by a team of linguists between 1990 and 2007, for a set of 147,278 unique words; thus, MI ‘knows’ little about special terms used by professional communities whereas LSA can target specific semantic spaces belonging to defined groups of speakers, such as in the business press or *PR Newswire* linguistic community.

More detailed descriptions of these algorithms may be found in the Methods section. Together, LSA and MI may cover multiple aspects of actual language usage. If these algorithms can significantly explain the observed correlations of surveys, it implies that the main source of variance in these surveys is language. This is problematic because semantics then determine the relationship between independent and dependent variables, as we will show below.

### ‘Leadership’, ‘motivation’ and ‘outcomes’ in semantics and in theory

Particularly salient examples of our theory are found in research on constructs such as ‘leadership’, ‘motivation’ and their purported outcomes. These constructs are prevalent in the research field known as Organisational Behaviour (OB), where central research topics are different types of leadership and their relationship to psychological processes in workplace behaviours. One of the most well-researched and popular theories of leadership during the recent decades has been ‘transformational leadership’, belonging to a set of leadership theories called ‘neo-charismatic leadership’ [25]–[29]. In a thorough review of these neo-charismatic leadership theories, Van Knippenberg and Sitkin [30] outline several serious problems that render these theories inaccessible to empirical investigation. One such important problem is the conflation of cause and effect in both definitions and measurement models. There is no universally accepted definition of ‘leadership’ as a scientific construct, but most definitions and practical usages somehow imply the achievement of results. Insofar as organisational success is a definition of leadership, the construct remains tautological as both cause (independent variable) and effect (dependent variable) are semantically given through definitions and operationalisations.

We will argue that the analysis of Van Knippenberg and Sitkin is applicable to other theories of leadership as well, at least as long as surveys are used for measurement.

The concept of ‘motivation’ is a good case in point. An examination of the semantic network of ‘leadership’ in the lexical database WordNet shows how leadership is related not only to outcomes but also to motivation; some popular definitions of ‘leadership’ are precisely acquired through motivating people to rally around some objective [24], whereas ‘motivation’ is a general descriptor for incidents or states that elicit acts. Leadership is thereby more tightly linked to motivation than to outcomes, whereas motivation is linked equally to outcomes and leadership. Were one to create a structural equation of these relationships, one might argue theoretically and semantically that motivation should mediate the relationship between leadership and outcomes.

There are different ways to assure consistency in scales. Some scholars have argued that items should sample from a wide, non-synonymous domain to avoid semantically caused alpha coefficients [31]. This principle is sometimes adopted in personality tests. We therefore expect that our semantic theory may not apply to correlation patterns from a statistically firmly grounded personality test built on the Five-Factor Model [32].

Our proposal is therefore that the quantitative relationships among these variables as surveyed are largely determined by their semantic properties. The psychometric validation of latent variables usually depends on the following steps of statistical analysis:

1. Establishing that every scale used is coherent, using Cronbach's alpha or similar;
2. Verifying that the scales are semantically independent of each other, using factor analytic techniques;
3. Establishing a quantitative model of how the variables are related to each other, usually involving structural equation models or some kind of statistical ‘mediation’ effect signalling that the findings are part of a larger nomological network; and
4. Using various statistical procedures to establish fit indices, used to determine the statistical significance of the whole model as such, compared to contrasting explanations or chance.

In the following sections, we will show how all these steps may be replicable in four large different samples using language algorithms applied to state-of-the art survey scales on leadership, motivation, outcomes and personality. We hope thereby to show that new technologies may be developed to illuminate this field, and also to substantiate our claim that psychometric assumptions about the origin of quantitative variation need to be revised.

### Ethics statement

The following four sections all contain their own description of methods. We declare that for all of them, data from human subjects were collected according to the ethical regulations for doing research in Norway, where the data were obtained. All respondents consented to take part voluntarily and were informed that they could withdraw from the study at any time. This research is not health related, but only asks participants to anonymously fill out survey forms with non-sensitive questions. Norway has no specific ethics committee to oversee this kind of non-clinical research, but a governmental body called the Norwegian Data Protection Authority (NDPA) rules whether such projects must be approved and registered to guarantee the legal and ethical protection of participants. We asked NDPA about the data collection (inquiry no. 28024 in 2006). The NDPA ruled that anonymous participation, i.e. submitting a completed survey questionnaire, is taken as sign of consent in three of these studies, as the procedure was seen as harmless, the questions were deemed not sensitive, and there were no way of tracing either answers or non-compliance back to individual respondents. In Study 4, the NDPA stated that an official approval needed to be obtained, being the only data collection with personally related content (a personality test). This approval and the subsequent registering and overseeing of the project was done by the Norwegian Social Scientific Data Services, which is the organization entrusted by the authorities to carry out this task. The approval for Study 4 data collection was given in the year 2006 and most recently renewed l in 2012 under the project number 27022, ref no. 27022/3/MSS. Data from this study were collected after written consent, and the written consent is kept as a variable in the original dataset collected through a survey website. These practices are entirely compatible with Norwegian law, research ethics and the ethical principles of the institution where the data were collected. The procedure is approved by the NDPA which is the designated regulating body for non-clinical research involving human respondents.

## Methods

### LSA algorithm calculations

Only an overview is given of the four core steps of the LSA process because of the careful treatment of LSA elsewhere, including a publication by one of the authors [33].

#### Step 1. Preparing term-document matrix

LSA starts by creating a term-document matrix, A, containing a weighted count of how many times a word, i, appears inside a document, j. The weighting method employed here, log-entropy, has been found generally to outperform other LSA weighting schemes [17], [34].

#### Step 2. Creating semantic space

After appropriate preparation (weighting, normalisation, etc.), this matrix is decomposed using ‘singular value decomposition’, a mathematical algorithm similar to a factor analysis, with the result being a semantic space of a given dimension represented as three matrices: U, a term-by-dimension matrix representing words; S, a singular value matrix; and V, a document-by-dimension matrix representing documents. The equation can be written as:
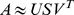
where U and V are orthogonal matrices whereas S is a diagonal matrix with main diagonal entries sorted in decreasing order. In practice, A could be approximated with 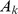 by preserving the first k singular values and the corresponding first k columns in U and V. The approximation can be written as:
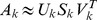
where 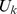 is a term-by-k matrix, 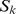 is a k-by-k matrix and 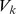 is a document-by-k matrix. This approximation estimates A with minimal error and also translates the term-by-document matrix into a correlated semantic space. Thus, each row vector of 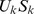 represents a word in the semantic space and has k columns which give the vector of the word in the semantic space. Likewise, each row of 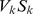 represents a document vector that correlates topics in the semantic space. By preserving the first k diagonal elements in S, the low-rank approximation produces the mutual constraints among words in different documents.

#### Step 3. Projecting items into the semantic space

Given the query *q*, which is a survey item, query vector *q* is obtained through an aggregation of word vectors relevant to the item. In our research, every item is projected into the semantic space as a query vector, 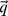, and that vector is saved as 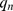 for future item-item analysis, where n is the total number of items.

#### Step 4. Calculating the similarity of items

To find similar items to 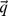 the query vector is then compared against all the items stored inside the semantic space, 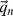, using the cosine similarity measurement, where n is the total number of stored items:
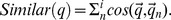



### MI algorithm calculations

The MI sentence similarity measure is computed for two candidate sentences, S_1_ and S_2_, as follows.

#### Step 1. Identify part-of-speech (POS)

The process begins with tokenisation and POS tagging of all the words in the survey item with their respective word classes (noun, verb, adverb, adjective and cardinal, which also plays a very important role in text understanding).

#### Step 2. Calculate word similarity

Each word in the sentence is measured against all the words from the other sentence to find the highest semantic similarity (*maxSim*) from six word-similarity metrics originally created to measure concept likeness rather than word likeness. The metrics are adapted here to compute word similarity by computing the shortest distance of given words' synsets in the WordNet hierarchy. Word-word similarity is computed only on words from the same word class, which are either from noun or verb word classes because WordNet contains separate semantic trees for nouns and verbs. Thus, it is not possible to obtain similarity between nouns and verbs using WordNet distance. For other word classes such as adverb, adjective, cardinal, and unknown words, whole-word matching is used instead. The word-word similarity measure is directional. It begins with each word in S_1_ being computed against each word in S_2_ and then vice versa.

#### Step 3. Calculating sentence similarity

Once the highest semantic similarity (*maxSim*) for each word in the sentences is computed, it is normalised by applying ‘inverse document frequency’ (*IDF*) to the British National Corpus to weight rare and common terms. The normalised scores are then summed up for a sentence similarity score, *Sim_MI_*, as follows:
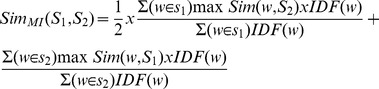
where *maxSim*(*w*, *S_2_*) is the score of the most similar word in *S_2_* to *w* and *IDF* (*w*) is the IDF of word *w*.

### The problem of signs

Neither LSA nor MI discriminates well between negative or positive assertions, and MI does not take negative values at all. The two sentences “It is raining” and “It is not raining” are indexed as very similar with high positive semantic scores, and both of them are very different from “The cat is on the mat”.

The handling of positive and negative values is of principal importance in the following analysis, and we need to dedicate some attention to this issue. The relationship between two series of numbers depends greatly on the distribution of signs. Appropriate handling of the direction (sign) of the correlations is crucially important to estimating the true mutual variance between semantic similarity indices and observed survey correlations.

In the case of backward-scored items, to prevent biases from response sets in the respondents, these are easily corrected. However, one of the surveys we apply here–the Multifactor Leadership Questionnaire (MLQ, see further explanation below)–does not contain such scores. Within this survey, 264 (26.7%) of 990 pairs of items are negatively correlated. Theory suggests that two scales, ‘laissez-faire’ and ‘passive management by exception’, are likely to relate negatively to effective leadership. Common sense seems to indicate that pairing items from these scales with items from other scales would correlate negatively. One typical example of negative correlation is between a) an item stating that a manager is unapproachable when needed and b) another item stating that the same person uses appropriate methods of leadership. The surveyed responses to these items correlated −.42 in our sample, but the semantic identity values range between .38 and .75. There is no *a priori* reason to assume that these correlations should be positive. Theory predicts this variance, in the way one may use one-tailed significance tests if there is no reason to assume both-way variation. Based on this, we allowed the signs of semantic identity scores to be negative for all pairs of items from ‘laissez-faire’ and ‘passive management by exception’ (except from among themselves), using available theoretical knowledge even before beginning the empirical survey (correctly identifying 255 of the 264 negative correlations, p<.001).

## Study 1

### Measures and sample

We chose the Multifactor Leadership Questionnaire (MLQ) due to its widespread use and central position in leadership research over the recent decades [35]. It was administered to 1,649 respondents from geographically dispersed units of a financial company. For 424 (25%), no demographic characteristics were available. Among those whose background was known, 51.1% were males, and the average age was 46 years (SD = 11 years). Two percent belonged to top management, 26% were middle managers, and 71% did not hold management positions. All participants rated their immediate supervisor. The survey was conducted in Norway, using a Norwegian translation of the MLQ.

### Results

Table 1 shows the alpha values of the MLQ as computed from the surveyed data and semantic similarity indices. The first column shows alphas obtained using survey responses. The middle column displays alphas computed from the MI values alone, which shows the internal consistency of the purely semantic representations of item pairs. The rightmost column show alphas computed from predicted correlations, i.e. the item-pair correlations saved as predictions when regressing semantic scores on the observed scores. These alphas would be those obtained if we use correlations predicted by semantic algorithms. Of 12 comparisons, in only one case did the semantically predicted alphas fall into a different tolerance range from those empirically observed. Despite the low number of items in each scale (3 to 5), all but one of the semantically predicted alphas fell into the acceptable or good ranges [31], and the survey-score alpha for the exception was also insufficient. The magnitudes of the survey-obtained alphas correlated .56 with the semantically predicted alphas (one-tailed p<.05, N = 12).

**Table 1:** Alpha values for the MLQ subscales, by surveyed and semantically obtained data.

	Cronbach's α by source of data		
MLQ scale	Empirically observed α	MI semantic α	α from semantically predicted correlations
Idealised influence attributes	0.85	0.82	0.80
Idealized influence behavior	0.87	0.79	0.81
Inspiring motivation	0.61	0.45	0.51
Intellectual stimulation	0.87	0.79	0.79
Individualized consideration	0.88	0.85	0.83
Conditional reward	0.83	0.82	0.81
Mgmnt by exception active	0.72	0.85	0.83
Mgmnt by exception passive	0.60	0.83	0.81
Laissez-Faire	0.83	0.83	0.80
Extra effort	0.89	0.77	0.78
Effective group	0.76	0.84	0.84
All outcomes	0.92	0.90	0.91

We regressed the semantic similarity indices on the empirically observed correlations among the MLQ items, which yielded an R^2^ of .79, p<.01. Finally, we computed a General Linear Model (GLM) with: a) MLQ correlations as dependent; b) semantic indices as covariates; and c) knowledge about scale belongingness as a fixed variable (making complete use of the knowledge that is accessible prior to running a survey). This model yielded an R^2^ of .86, p<.01. For both regression models, we saved the predicted values and residuals. Table 2 compares the actually observed mean correlations with those predicted in the two regression models. The table shows that linear regression predicts the observed values remarkably well, with the exception of ‘Mgmnt by exception active’. The predicted values from the GLM were precisely identical to the observed correlations (in this case, N equals the number of item pairs, in this case 990 unique item pairs).

**Table 2:** Observed average correlations between the single scales of the MLQ and the items measuring outcomes, compared with the correlations predicted in linear regression by semantic similarity scores.

MLQ scales with outcome variables	Average surveyed correlations	Linear regression predicted correlations	GLM predicted correlations
Idealised influence (attrib.) with outcome	.52	.45	.52
Idealised influence (beh.) with outcome	.51	.44	.51
Inspiring motivation with outcome	.52	.47	.52
Intellectual stimulation with outcome	.50	.43	.50
Individualised consideration with outcome	.54	.48	.54
Conditional reward with outcome	.47	.43	.47
Mgmnt by exception active with outcome	.16	.42	.16
Mgmnt by exception passive with outcome	−.19	−.25	−.19
Laissez-faire with outcome	−.36	−.25	−.36
Outcome with outcome	.60	.53	.60
Random pairs of items	.18	.19	.18

GLM predicted scores in the rightmost column.

CFA of all 10 MLQ subscales in the present sample yielded a comparative fit index (CFI) of .93, a root mean square error of approximation (RMSEA) of .05, and a standardized root mean square residual (SRMR) of .07. The error terms were not correlated, and these figures are usually interpreted as indicative of an acceptable model [36].

## Study 2

### Measures and sample

Again, the first 36 items describing leadership behaviours from the MLQ [35] were administered along with seven items measuring economic-exchange perceptions, eight items measuring social-exchange perceptions [37], and six items measuring intrinsic motivation [38]. The outcome variables were the additional nine outcome measures from the MLQ, seven items measuring organisational citizenship behaviour (OCB) [39], five items measuring turnover intention (TI) [40], and self-rated work quality and work effort, each measured by five items [38].

The sample consisted of 255 employees at a governmental research agency, mostly scientists and engineers. Of these, 66.7% were male, and the mean age was 38 years. One quarter rated themselves as managers, and the rest termed themselves as “project team members”.

### Results

Using observed correlations, the alphas ranged from .53–.96, mean = .85. For the semantic alphas, these numbers were .58–.97, mean = .86, and for the predicted correlations, .35–.91, mean = .72. These latter values correlated (.91 and .92) with the alphas obtained empirically (p<.01).

We regressed the semantic similarity indices on the observed set of inter-item correlations, obtaining an adjusted R^2^ of .53 (p<.01). A second analysis applied a GLM model with information about scale belongingness, bringing the adjusted R^2^ to .68 (p<.01).

Saving the predicted values and residuals from the regression equations, Table 3 lists all relationships from leadership to motivation, from motivation to outcomes, and from leadership behaviours to outcomes. As in the previous study, linear regression alone predicted the relationships among the scales in the survey (rho = .90, p<.01), and when using the GLM approach, the predicted values again become identical to the observed correlations from human survey respondents.

**Table 3:** Relationships between leadership behaviours, motivation and outcomes as rated by the MLQ.

Main construct relationships	Scale relationship	Average observed correlations	Average correlations predicted from linear regression	GLM predicted correlations
***Leadership to motives:***	Transformat. leadersh.→Economic exchg.	−.10	−.07	−.10
	Transformat. leadersh.→Intrinsic motiv.	.18	.15	.18
	Transformat. leadersh.→Social exchg.	.15	.11	.15
	Transactional leadersh.→Economic exchg.	.01	.01	.01
	Transactional leadersh.→Intrinsic motiv.	.03	.08	.03
	Transactional leadersh.→Social exchg.	.05	.06	.05
	Laissez-faire→Economic exchg.	.11	.17	.11
	Laissez-faire→Intrinsic motiv.	−.11	−.07	−.11
	Laissez-faire→Social exchg.	−.07	−.03	−.07
***Motives to outcomes:***	Intrinsic motiv.→OCB	.20	.24	.20
	Intrinsic motiv.→Turnover int.	−.22	−.16	−.22
	Intrinsic motiv.→Work effort	.26	.24	.26
	Intrinsic motiv.→Work quality	.21	.22	.21
	Social exchg.→OCB	.12	.18	.12
	Social exchg.→Turnover intent.	−.14	−.08	−.14
	Social exchg.→Work effort	.13	.15	.13
	Social exchg.→Work quality	.05	.16	.05
	Economic exchg.→OCB	−.15	−.19	−.15
	Economic exchg.→Turnover int.	.13	.23	.13
	Economic exchg.→Work effort	−.17	−.15	−.17
	Economic exchg.→Work quality	−.09	−.14	−.09
***Leadership to outcomes:***	Transformat. leadersh.→OCB	.10	.16	.10
	Transformat. leadersh.→Turnover int.	−.16	−.07	−.16
	Transformat. leadersh.→Work effort	.09	.15	.09
	Transformat. leadersh.→Work quality	.07	.16	.07
	Transactional leadersh.→Turnover int.	.05	.08	.05
	Transactional leadersh.→Turnover int.	−.07	.02	−.07
	Transactional leadersh.→Work effort	.06	.08	.06
	Transactional leadersh.→Work quality	.07	.08	.07
	Laissez-faire→OCB	−.01	−.09	−.01
	Laissez-faire→Turnover int.	.11	.16	.11
	Laissez-faire→Work effort	−.03	−.09	−.03
	Laissez-faire→Work quality	.01	−.08	.01

Observed correlations and values obtained through semantic analysis.

**Figure 1:**
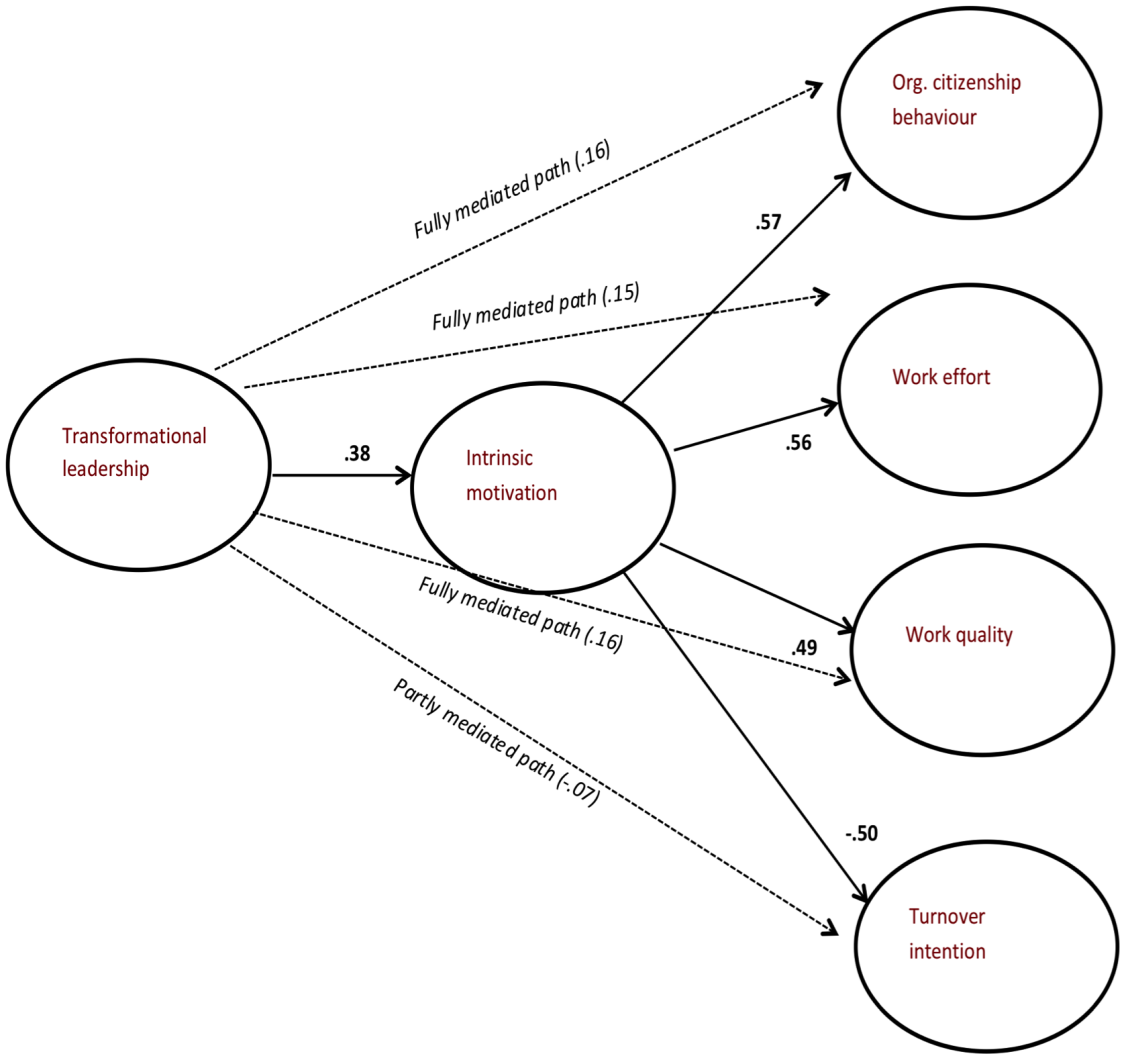
Direct and “mediated” semantic relationships between transformational leadership, intrinsic motivation and organizational outcomes (direct semantic relationships from transformational leadership to outcomes in brackets).

To explore how semantic values can explain claims of ‘mediation’ among survey variables, we ran hierarchical regression analyses with the organisational-outcome variables as dependent variables: Transformational leadership as independent in Step 1, and intrinsic motivation as independent in Step 2. Satisfying criteria for mediation, transformational leadership significantly predicted work effort, work quality, OCB, and TI in the first step (p<.01), but these relationships were rendered insignificant when intrinsic motivation was added in the analysis. Table 4 shows the aggregated-level correlations among the variables and lists the results of the mediation analysis. We mapped this situation using *only* semantic information in Figure 1. As can be seen, the semantic values fit the pattern compatible with mediation.

**Table 4:** Correlations between transformational leadership, intrinsic motivation and outcome variables, with tests of mediating relationships from hierarchical regression.

Variables	Transf. Leadership	Intrinsic motivation	Mediated by intrinsic motivation:
Intrinsic motiv.	.32**		
Work effort	.17**	.42**	Fully
Work quality	.13*	.33**	Fully
Org. Citizen. Behav.	.19**	.33**	Fully
Turnover Intention	−.30**	−.35**	Partly

**Correlation is significant at the .01 level (2-tailed).

*Correlation is significant at the .05 level (2-tailed).

This dataset contains 16 individual scales. Ideally, a CFA should identify all of them with good fit. That did not happen, as a CFA for 16 factors returned a CFI of .82, a RMSEA of .05 and an SRMR of .07. If one allows the MLQ to be left out, the indices improve to a marginal fit (CFI = .89, RMSEA = .06, SRMR = .06), indicating some cross-loadings among items [36].

## Study 3

### Measures and sample

This study compared semantic indices with responses to a broad range of leadership and motivation scales. Transformational leadership was measured with the 20 items from the MLQ [35]; leader-member social exchange was used to measure leader-member-exchange (LMX) [41]; and the Ohio State Leadership 2-factor theory was assessed by a subsample of 10 items measuring initiation of structure and 10 items measuring consideration from the Leadership Behavior Development Questionnaire (LBDQ) [42].

We used eight items of affective organisational commitment published by Meyer, Allen, and Smith [43] and three items measuring job satisfaction published by Cammann, Fichman, Jenkins, and Klesh [44]. The outcome variables were TI [40], self-rated work quality and work effort, each measured by five items [38]. These scales were administered as a Norwegian-language web-based survey of 981 civilian and military employees in the Royal Norwegian Armed Forces. No demographics were available in the latter sample, but they reflect a random selection of military professionals, with a majority of males and a mean age somewhere in the 30s. Most respondents are likely to be military officers and thus to have had personal leadership training and experience.

### Results

Alphas from observed correlations ranged from .86–1.00, mean = .94. For semantic alphas, these numbers were .84–.99, mean = .94 and for the predicted correlations .71–.99, mean = .89. These latter values correlated (.88 and .72) with the alphas obtained through the survey (p<.01).

Regressing the semantic similarity indices on the observed correlations, we obtained an adjusted R^2^ of.47 (p<.01). When scale belongingness was entered as a fixed factor in GLM, the adjusted R^2^ was .87 (p<.01). The predicted values and residuals were saved and are displayed in Table 5, showing the mean correlations among leadership variables, motivational states and outcome variables, along with the predicted values from the two types of regression equations. Again, the predicted values from GLM were identical to the observed correlations.

**Table 5:** Relationships among three theoretical leadership models, motivation and outcomes, using empirically surveyed correlations and correlations predicted by semantic values.

Main construct relationships	Scale pairs	Mean observed correlations	Predicted correlations in lin. regression	Correlations predicted in GLM
**Leadership to Leadersh.**	Consideration → Consideration	.55	.29	.55
	Consideration → Initiate struct.	.23	.32	.23
	Consideration → LMX	.47	.28	.47
	Consideration → Transform. lead.	.47	.29	.47
	Initiate struct. → Initiate struct.	.33	.34	.33
	Initiate struct. → LMX	.27	.29	.27
	Initiate struct.→Transform. lead.	.34	.31	.34
	LMX→LMX	.63	.37	.63
	LMX → Transform. lead.	.47	.27	.47
	Transform. lead. →Transform. lead.	.56	.29	.56
	**Mean absolute values**	**.43**	**.31**	**.43**
**Leadership to Motives**	Consideration → Affective comm.	.21	.34	.21
	Initiate struct. → Affective comm.	.13	.35	.13
	LMX- > Affective comm.	.20	.31	.20
	Transform. lead. → Affective comm.	.22	.30	.22
	Consideration → Job sat.	.36	.31	.36
	Initiate struct. → Job sat.	.19	.34	.19
	LMX → Job sat.	.33	.32	.33
	Transform. lead. → Job sat.	.32	.30	.32
	**Mean absolute values**	**.24**	**.32**	**.24**
**Leadership to Outcomes**	Consideration → Turnover int.	−.26	−.17	−.26
	Consideration → Work effort	.16	.31	.16
	Consideration → Work quality	.11	.32	.11
	Initiate struct. → Turnover int.	−.13	−.20	−.13
	Initiate struct. → Work effort	.12	.35	.12
	Initiate struct.→ Work quality	.10	.34	.10
	LMX → Turnover int.	−.24	−.17	−.24
	LMX → Work effort	.15	.32	.15
	LMX → Work quality	.14	.31	.14
	Transform. lead. → Turnover int.	−.23	−.16	−.23
	Transform. lead. → Work effort	.17	.30	.17
	Transform. lead.→ Work quality	.14	.32	.14
	**Mean absolute values**	**.16**	**.27**	**.16**
**Motive to Motive**	Affective comm. → Affective comm.	.43	.40	.43
	Affective comm. → Job sat.	.40	.34	.40
	Job sat. → Job sat.	.68	.45	.68
	**Mean absolute values**	**.50**	**.40**	**.50**
**Motive to Outcome**	Affective comm. → Turnover int.	−.37	−.20	−.37
	Affective comm. → Work effort	.22	.33	.22
	Affective comm. → Work quality	.14	.34	.14
	Job sat. → Turnover int.	−.49	−.22	−.49
	Job sat. → Work effort	.31	.38	.31
	Job sat. → Work quality	.17	.36	.17
	**Mean absolute values**	**.28**	**.31**	**.28**
**Outcome to Outcome**	Turnover int. → Turnover int.	.62	.38	.62
	Turnover int. → Work effort	−.15	−.22	−.15
	Turnover int. → Work quality	−.08	−.22	−.08
	Work effort → Work effort	.54	.42	.54
	Work effort → Work quality	.35	.36	.35
	Work quality → Work quality	.48	.41	.48
	**Mean absolute values**	**.37**	**.33**	**.37**

The data from a CFA of this full dataset displayed the following values: CFI was .85, the RMSEA was .06 and the SRMR was .06. The considerable cross-loadings were expected due to the conceptual overlap of many of the scales and individual items included in this study.

## Study 4

### Measures and sample

We used an officially translated, Norwegian version of a commonly used five-factor model inventory called the NEO-FFI (the name NEO stems from the three first factors, ‘neuroticism’, ‘extraversion’ and ‘openness’) [32], [45]. The FFI-version of the NEO is a short form, which we administered using a web-based survey form to 5,332 students from a leading business school in Norway. The mean age was 25 years, and 44.7% were male. This version has 60 items, yielding a total of 1,770 unique pairs of item correlations.

### Results

The alphas of the NEO-FFI ranged from .94 to .96, which are considered excellent [31]. Alphas computed by semantic values (MI) ranged .37–.88 with a mean of .64, which we considered questionable. The semantically obtained alphas for ‘extraversion’ and ‘neuroticism’ were not bad (.88), but the three others were much lower and not satisfactory.

Regressing the semantic similarity indices on the empirically obtained correlation values as the dependent variable, we found an R^2^ of only .004 (p<.05). The model as such reached only marginal significance. The saved and predicted values from the regression did not produce any recognisable patterns, see Table 6.

**Table 6:** Scale relationships in the five factors of the NEO-FFI, observed survey correlations and semantically predicted values.

Scale	Observed correlations	Correlations predicted in linear regression
A→A	.18	.05
C→A	.05	.04
C→C	.28	.05
E→A	.04	.04
E→C	.09	.05
E→E	.23	.05
N→A	−.02	.04
N→C	−.10	.05
N→E	−.10	.05
N→N	.21	.05
O→A	.02	.05
O→C	.03	.05
O→E	.05	.05
O→N	.00	.04
O→O	.20	.05

**Figure 2:**
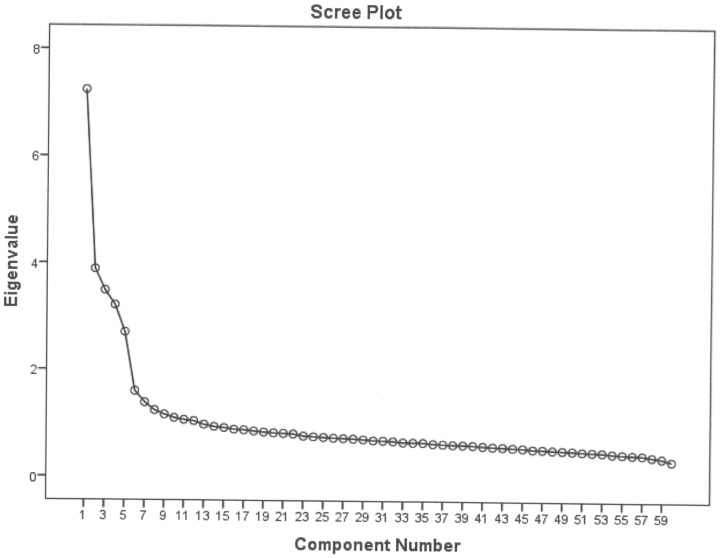
Scree plot of the NEO-FFI.

The scree plot from an exploratory factor analysis of the survey data indicated the usual five factors very clearly in the sample responses (see Figure 2). A CFA expecting five factors in this dataset showed a poor CFI (.85) but good RMSEA (.04) and SRMR (.03). The theoretically assumed five factors were obviously present in the data, but not well detected by either semantics or factor analysis.

## Discussion and Implications

Applying the text algorithms LSA and MI to a wide range of survey scales commonly used in management research, we were able to significantly explain the major part of variation in surveys. The correlations we predicted in multiple regression were similar to those created by human respondents. Allowing the algorithms to ‘know’ the context in GLM, we actually obtained correlations identical to those of human subjects. We were able to show that semantic relations not only predict the intra-scale coherence measured by Cronbach's alpha, but also the observed correlation relationships and proposed ‘mediating’ relationships among the variables. The factor-analytical fit indices were generally better the more semantics seemed to determine the correlation matrix. In this sense, CFA did not detect or prevent the pervasive influence of semantic commonalities. In fact, our results indicate that constructing a survey on mere semantic relationships among the items is an easy way to obtain good fit indices in CFA.

The personality test results in our study were not significantly explained by semantics. We expected this, since personality test scores are constructed to vary more freely, but still reflect the underlying construct and allow differentiated descriptions of people. This also shows that the long-proposed “lexical hypothesis” [46] in personality research has no immediate relevance to a STSR. While it is highly unlikely that our results were due to chance, one should bear in mind that the text analysis algorithms are steadily developing. Future algorithms are likely to make more advanced use of available information, creating even better and more differentiated estimates than ours. As explained earlier, the simplicity and well-documented functioning of the selected semantic algorithms strengthens the findings of this research.

Psychometric principles for construct validation seem, at least in their present form, as frequently applied in organisational psychology, to need revision to incorporate our findings. The semantic properties seem to pervade survey responses throughout many parts of the data analysis from the alpha coefficients to the CFA. This represents a fundamental problem to the understanding of psychometric principles in scientific research. Our study shows that the relationship between independent and dependent variables may be semantically determined a priori to conducting the survey since it follows from the wording of the items. This is in accordance with the previous theoretical analysis of van Knippenberg and Sitkin [30]. A more troubling finding is that this confounding of variables was not restricted to leadership, but appeared in other OB measures as well, such as motivation, job satisfaction, and work outcomes. It also affected the relationships between surveys from different leadership theories, casting doubt on the claims that some of these matter more than others [47], as they are simply different ways of stating the same propositions.

At present, it is difficult to assess the pervasiveness of the problems we have detected here. In our study, all the commonly used measures from the field of OB were substantially affected by semantics, whereas the personality test showed very little influence. It is possible that some social scientific concepts are more abstract than others, thus being rendered more vulnerable to mere semantic relationships. It has been known for some years that common method variance usually leads to more inflated statistics in this field than in other fields [9], [48]–[50]. The phenomenon we have detected here may be less problematic in other disciplines. The core of the problem seems to be an uncritical assumption that statistical methods separating signal from noise in survey responses are sufficient to ascertain the objective existence of a construct, a practice that has been criticised on theoretical grounds [51]–[54]. It may be that survey responses collecting less abstract responses, grounded on observations of behaviour instead of cognition [55], [56], are less prone to semantic calculations of the kind demonstrated here.

But ultimately, the only way of ruling out semantic influences as a major source of co-variation in survey data is to identify this influence in advance. Relationships among surveyed variables are commonly tested with 0-hypothesis statistics, implying the expectations that survey items are randomly related. Our findings instead suggest that all items are likely to be related though semantic commonalities. Perhaps replacing the 0-hypothesis with the semantic hypothesis is a more solid way of separating the empirical information from a merely semantic relationship in surveys.

Our findings have the following major implications:

1. Technologies for digital text analysis have advanced to a point of offering important and interesting usages to the social sciences. Text algorithms and similar procedures play an important role in indexing, storing and developing knowledge in the social sciences. Such knowledge is already in use for industrial purposes and we are taking it some steps further into the field of psychological research, such as OB. We believe this is now emerging as a promising field with many possible applications in our increasingly digitalised scientific society.
2. The fact that surveys seem predictable before the questioning of real subjects seems bewildering to many. And yet, our data show beyond reasonable doubt that this is possible. This opens up opportunities for experimental research on people's ability to understand logical propositions and our capacity to differ between logical and empirical statements. As shown in research on cognition, advanced forms of thinking in humans is an energy-consuming and partially unpleasant activity [57]. The semantic network of language may function as a guide for thinking that creates uniform effects on most speakers, but with little meta-cognition in the speakers themselves.
3. Cross-cultural research using surveys needs to be re-examined in view of the present findings. Our findings were obtained using semantic similarity indices computed in American English, regressed on scores obtained from surveys in Norwegian. As long as the results are explained by semantics, all we can know about survey results being similar across cultures was that the survey was correctly translated. This pertains directly to the relationship among logical and empirical propositions: While the same propositions may be stated in different languages, their empirical implications in terms of behaviour dynamics may not be the same.
